# Analogy, Cognitive Architecture and Universal Construction: A Tale of Two Systematicities

**DOI:** 10.1371/journal.pone.0089152

**Published:** 2014-02-25

**Authors:** Steven Phillips

**Affiliations:** Mathematical Neuroinformatics Group, Human Technology Research Institute, National Institute of Advanced Industrial Science and Technology (AIST), Tsukuba, Ibaraki, Japan; Utrecht University, Netherlands

## Abstract

Cognitive science recognizes two kinds of systematicity: (1) as the property where certain cognitive capacities imply certain other related cognitive capacities (Fodor and Pylyshyn); and (2) as the principle that analogical mappings based on collections of connected relations are preferred over relations in isolation (Gentner). Whether these kinds of systematicity are two aspects of a deeper property of cognition is hitherto unknown. Here, it is shown that both derive from the formal, category-theoretic notion of universal construction. In conceptual/psychological terms, a universal construction is a form of optimization of cognitive resources: optimizing the re-utilization of common component processes for common task components. Systematic cognitive capacity and the capacity for analogy are hallmarks of human cognition, which suggests that universal constructions (in the category-theoretic sense) are a crucial component of human cognitive architecture.

## Introduction

Cognitive science recognizes two kinds of systematicity. One kind of systematicity is the property of cognition where the capacity for certain cognitive abilities implies the capacity for certain other cognitive abilities, i.e. capacity is distributed around equivalence classes of cognitive abilities [1]. Another kind of systematicity is the preference for analogical mappings based on collections of connected relations over relations in isolation [2]. Whether these two kinds of systematicity are aspects of a deeper property of cognition is hitherto unknown.

In previous work [3]–[6], we explained Fodor and Pylyshyn's kind of systematicity using the category theory notion of *universal construction* [7]. The new aspect of the current paper is the use of universal constructions to also explain systematicity in the context of analogical mapping. Here, the two kinds of systematicity are recalled in the remainder of this introduction before a category theory account of both is provided in the subsequent sections.

### Systematicity (Fodor and Pylyshyn)

A remarkable property of human cognition is the distribution of cognitive capacities, where the capacity for certain cognitive abilities implies the capacity for certain other cognitive abilities. For example, suppose one is shown pairs of geometric shapes such as a square to the left of a triangle. If one has the ability to infer square as the left shape in the pair (square, triangle), then one also has the ability to infer triangle as the left shape in the pair (triangle, square), assuming that squares and triangles are individually recognizable. This property is generally referred to as *systematicity* [1], and is characterized more broadly as having capacity 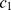 *if and only if* 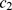 [8], i.e. as equivalence classes of cognitive capacities.

For cognitive science, a major challenge has been to provide a theory of cognitive architecture that *explains* systematicity: i.e. *why* certain cognitive capacities are distributed in a particular, non-arbitrary way [1], [9], [10]. Cognitive architecture is the collection of basic processes and their modes of composition that together provide the basis for a slew of cognitive abilities, from recognizing concrete physical objects to reasoning about abstract mathematical structures. The problem with the major theoretical frameworks, including classicism (symbols systems) and connectionism (neural networks), is that systematicity does not necessarily follow from the core principles and assumptions of their proposed theories. The essential problem is that although classical and connectionist theories are sufficiently general to derive models supporting the systematicity property (in the cognitive domains of interest), they are insufficiently specific to rule out models (derived from the same core principles and assumptions) that do not support systematicity (in those same cognitive domains). Further, *ad hoc* (essentially, arbitrary) assumptions are needed that take up the explanatory slack to exclude those classical or connectionist models that do not support systematicity, and so neither classical nor connectionist theory fully explains systematicity [10].

The reasons for failure stem from postulated notions of compositionality: i.e. the ways in which a representation of a complex entity is constructed from the representations of the complex entity's constituents. The classical account of cognitive architecture is that cognitive processes are sensitive to *grammatical* structure such that the representations of complex entities are *tokened* whenever the representations of their constituents are tokened [1]. Thus, the two shape capacities (above) are inseparable because they involve a common process: say, 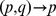, where 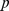 and 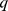 are symbols for squares and triangles, under the assumption of having component processes for recognizing squares and triangles. In other words, the presence or absence of the common grammatical rule implies the presence or absence of the entire group of cognitive (shape-inferring) capacities; under this compositional scheme, there is no case of having some, but not all capacities.

Similarly, a connectionist account of capacity can make use of common processes in the form of activation units and weighted connections modeling cognitive processes that are sensitive to *spatial* structure. In this case, the two shape capacities factor through a common (sub)network of weighted connections that map (neuronal) vector representations of shape pairs to first shapes, so that the presence or absence of this common network of connections implies the presence or absence of the collection of capacities.

Classical and connectionist models can be constructed such that capacity 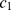 if and only if 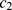, however, systematicity doesn't *necessarily* follow from classical, or connectionist theory, because one can also devise models from those theories such that 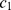 but not 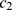 [10]. For example, the following instance of a classical (production rule) architecture: 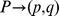, 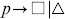, and 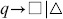 generates all four representations of square/triangle pairs; but the instance: 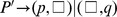, 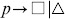, and 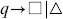 does not generate the pair 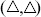, even though it can generate the other three (adapted from [3]). Connectionist theory also admits systematic and nonsystematic models in an analogous way, though the mode of compositionality may differ (see [11]). Additional (ad hoc) assumptions are needed to provide models with the systematicity property. (Ad hoc assumptions are like having arbitrarily many free parameters to fit data.) So, classical and connectionist theories fail to fully explain systematicity [10].

Classicists have rebuffed the claim that, by assuming only the systematic grammars, the classical explanation for systematicity relies on ad hoc assumptions [8]: accordingly, the Classical (Language of Thought, LOT) theory of cognitive architecture postulates only those grammars that generate “… all and only the mental sentences whose meanings are the contents of propositional attitudes that the cognizer has the ability to have. That the symbol system has such a grammar is not an auxiliary hypothesis that is *independent* of the LOT hypothesis.” ([8], footnote 16, original emphasis). Cognitive architecture is said to consist of grammars that afford a “canonical decomposition” of mental sentences (see [12], section 6.3), which would seem to rule out the example of a nonsystematic classical architecture given above. Yet, even if granted these assumptions (as not being ad hoc in nature), the principle that guarantees such grammars remains unspecified. Ironically, although classicists postulate a *representational/computational theory of mind*, following Turing [12], computer scientists have long recognized the idiosyncratic (ad hoc) nature of syntax in developing a theory of computation [13], and thus have turned to category theory as an approach to computational structure to obviate this problem (see, e.g., [14]–[17]). Our approach to systematicity is motivated analogously: essentially, the systematic constructions are the universal/optimal constructions, in a formally precise sense to be specified later.

### Systematicity (Gentner)

In the context of analogy, *systematicity* is the observation that when matching source and target domains of (relational) knowledge people match systems of (higher-order) relations in preference to isolated (lower-order) relations [2]. This observation is embodied as the *systematicity principle*, or assumption, in the *structure mapping theory* of analogy [2]. Structure mapping theory supposes that relational knowledge consists of a system of concepts arranged in tree-like structures. Three kinds of concepts are distinguished: *objects*, *attributes* and *predicates*. Object concepts are things like *John*: they are constants, which are nodes in a concept tree from which there are no more branches. Attributes and predicates are concepts that express some proposition about other concepts. An attribute expresses a proposition about a single concept, e.g., *Male*(*John*) expresses the proposition that *John is a male*, and its concept tree structure has *Male* (attribute) as the root concept node and John (object) at its only branch. Predicates are concepts that express propositions about two or more concepts, e.g., *Loves*(*John*, *Mary*) expresses the proposition that *John loves Mary*, and its concept tree structure has *Loves* (predicate) as the root node, *John* at its first branch and *Mary* at its second branch. Later, we consider objects, attributes and predicates as instances of relations of arity 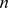: i.e. nullary (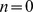), unary (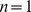) and *n*-ary (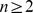) relations, respectively.

Predicates can express propositions about other predicates. For example, *Knows*(*Tom*, *Loves*(*John*, *Mary*)) expresses the proposition that *Tom knows that John loves Mary*. Its concept tree consists of the predicate *Knows* at the root with *Tom* at the first branch and the concept tree corresponding to the proposition that *John loves Mary* at the second branch. Predicates that express propositions about objects are called *first-order* predicates, and those expressing propositions about predicates are called *higher-order* predicates. In the current example, *Loves* is a first-order predicate and *Knows* is a higher-order predicate.

The Water-Heat flow analogy example (see [2]) illustrates the systematicity principle in analogical mapping. Suppose the following relational knowledge (concept trees) from the water flow domain:

1. *And(Contains(Vessel, Water), On-top-of(Lid, Vessel)); and*
2. *Cause(And(Puncture(Vessel), Contains(Vessel, Water)), Flows-from(Water, Vessel)).*

Suppose, also, two corresponding trees from the heat flow domain by replacing the objects in the water flow domain with corresponding objects in the heat flow domain, as given by the following object concept mappings: 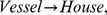 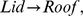 and 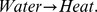 The systematicity principle as expressed in this example is the preference for mapping the second tree over the first, because the second tree involves a larger system of (higher-order) predicates than the first [2].

### Outline of approach

The novel aspect of this paper is two-fold: (1) a category-theoretic explanation for Gentner's kind of systematicity, and (2) an explanation for why these two kinds of systematicity are related via the category-theoretic notion of *universal construction*. In category theory, a universal construction relates a collection of arrows in some category of interest via a common *mediating* arrow in a unique way [7]. We used universal constructions to explain Fodor and Pylyshyn's kind of systematicity [3]–[6]. Intuitively, in a collection of systematically-related cognitive capacities (arrows), every capacity is composed of a common cognitive process (mediating arrow) in a unique way (no further, ad hoc, assumptions are needed) for the cognitive domain (category) of interest. Hence, the presence or absence of this common process implies the presence or absence of the collection of capacities.

Universal constructions can also be conceptualized as a kind of optimization: as the factorization of a collection of arrows via their greatest common arrow, which is analogous to the factorization of a collection of numbers via their *greatest common divisor*. This conception of universal construction as a form of optimization is the intuition behind a category-theoretic explanation for Gentner's kind of systematicity, where humans typically attempt to maximize the number of matches between source and target elements in an analogical mapping. Heuristically, the benefit of finding more matches is the greater transfer of knowledge from one (source) domain to facilitate inferences in another (target) domain.

The rest of this paper is concerned with detailing a category-theoretic link between these two kinds of systematicity. In the next section (Methods), we provide the basic category theory in regard to the universal constructions that are used to explain the two kinds of systematicity. Then, in the following section (Results), we derive these two kinds of systematicity from the category-theoretic notion of universal construction. The explanation for Fodor and Pylyshyn's kind of systematicity was already provided in previous work [3]–[6]. The essential points of this explanation are recalled here, but the presentation differs from the earlier work to facilitate the comparison with the explanation of Gentner's kind of systematicity. In the final section (Discussion), psychological/conceptual interpretations of these results are discussion, along with possible extensions to address some other aspects of analogy. The style of presentation in the main text is informal with the supporting technical details provided in the supplementary texts so that familiarity with category theory is not required.

## Methods

In this section, we provide an informal introduction to the category theory concepts needed to explain the two kinds of systematicity, i.e. universal constructions, which depend on the concepts of category and functor. An introduction to category theory typically starts with a definition of a *category*, which includes a collection of *objects* and relations between objects, called *arrows* (or *morphisms*, or *maps*). The category theory approach to cognition presented here regards a cognitive architecture as a category (of possibly other categories), where objects are interpreted as components of the architecture and arrows are relations between those components. For instance, two objects may be interpreted as two collections of representational states and an arrow between them as a cognitive process (function) mapping states to states. Hence, we begin our introduction from the more familiar notions of sets and functions between sets. The definition of a *universal construction* also depends on the concept of a *functor*, so we also provide an informal introduction to functors. Formal definitions are provided in the appendices. Deeper introductions to category theory can be found in many texts on the subject (e.g., [7], [18]).

### Category

A category is a collection of objects and arrows between objects with a composition operation for composing arrows to form new arrows in a way that satisfies certain axioms. They are the *associativity* and *identity* axioms (see Text S1). Many results in category theory apply at this abstract level, where the nature of the objects, arrows, and composition operator is left unspecified. More concretely specified categories are typically employed for particular domains of interest. For instance, we will consider the shapes example as part of a category whose objects are sets, arrows are functions between sets, and composition operation is function composition. More concretely still, some of these objects (sets) are sets of perceptual, or conceptual states for corresponding shapes, and the arrows are functions (cognitive processes) transforming representational states.

Objects and arrows may be constructed from other objects and arrows. The set of shape concept pairs, for example, is constructed from the *Cartesian product* of the set of shape concepts, 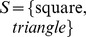, with itself: i.e. the set 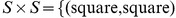,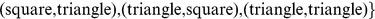, which is another object. The first and second elements of each pair are retrieved by two functions (also called projections): 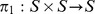; 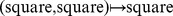, 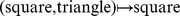, 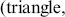 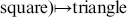, 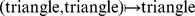; and 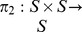; 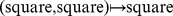, 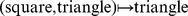, 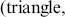 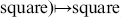, 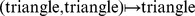. In general, the Cartesian product of sets 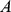 and 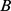 is the set 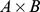 of all pairwise combinations of the elements taken from sets 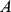 and 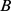, and two functions, 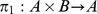 and 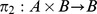, that return the first and second elements of each pair. A Cartesian product is a product in the category 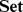. (Boldface is conventionally used for the names of categories.) More generally, in some category 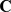, a product of objects 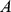 and 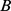 is an object 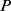 (also denoted 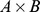) together with two arrows 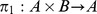 and 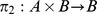 such that certain universal conditions are met, which will be specified when introducing the concept of universal construction.

### Functor

Functors are to categories as arrows are to objects. They send objects and arrows in one category to (respectively) objects and arrows in another category. Functors can also be considered as a way of constructing categories from other categories. For example, the *product functor* constructs product objects and arrows from pairs of objects and arrows. The product functor, in the context of 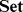, constructs the Cartesian product object 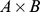 from objects 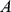 and 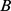, and the product function 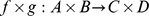, mapping pairs of elements, from the functions 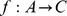 and 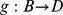. Universal constructions are defined with regard to functors.

### Universal construction

The intuition behind the formal notion of universal construction involves the idea of capturing the common component of a collection of entities (arrows). We can see this intuition in action from our shapes example. Observe that every pair of maps that extracts the first and second shape concept from shape images (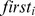 and 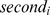) can be composed of a map sending each image to a pair of shape concepts in the Cartesian product set and the projections for extracting the first and second shape concepts from each pair of shape concepts. For example, the map 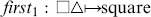 is composed of the map 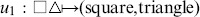 and the projection 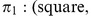 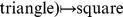. The map 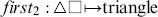 is composed of the map 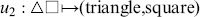 and 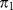. Maps 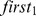 and 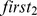 share the common component map 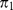. Similarly, maps that extract the second shape concept from each image, 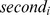, share the common component map 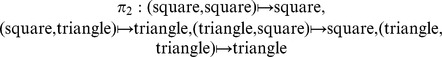.

Universal constructions can also be thought of as a kind of optimization relative to the underlying functor. In the case of products, the underlying functor of interest is the *diagonal functor*, which sends objects and arrows to pairs of objects and arrows. We will see in more detail later that every pair of arrows to the object 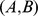 factors through the pair of projections 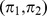 in a unique way.

## Results

Two kinds of systematicity are derived from universal constructions. For succinctness, Fodor and Pylyshyn's kind of systematicity is termed *F-systematicity*, and Gentner's kind of systematicity is termed *G-systematicity*.

### F-systematicity (Fodor and Pylyshyn)

The shapes example is used for the explanation of F-systematicity based on universal constructions. In this example of F-systematicity, if one has the capacity to infer from 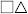 that the left shape is square, then one also has the capacity to infer from 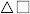 that the left shape is triangle, and likewise for the right shape in each instance.

Cognitive architecture is modeled in the category 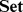 where objects are sets of cognitive representations, arrows are cognitive processes mapping representations, and the composition operator is function composition. For the specific shapes example, we have objects that are sets of representations of shape concepts (indicated by name, e.g., square) and images (indicated by symbol, e.g., 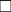), and arrows that are functions from representations to representations. For example, the set of shape concepts is the set 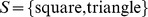, the set containing the square-triangle image is the *singleton* (one-element) set 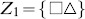, and the set containing the triangle-square image is the singleton set 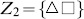. (We also have sets 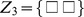 and 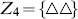.) The arrow representing the capacity to infer from 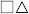 that the left shape is square is the function 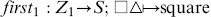, and the arrow representing the other left-shape inferential capacity is 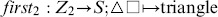. Likewise, we have arrows for right-shape inferential capacities: 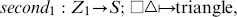 and 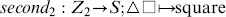.

F-systematicity follows from the fact that in 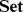 we also have the Cartesian product set of all pairwise combinations of elements of 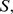 i.e. 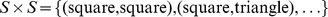, and two functions (projections) that return the first and second elements of each pair, i.e. 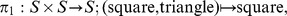 etc., and 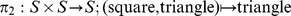, etc. Together, the Cartesian product and projections constitute the product construction 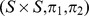, which is an instance of a universal construction. As a universal construction, for each set 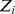 and each function 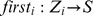, there must exist a unique function 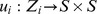 such that 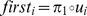 and 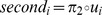. Indeed, for 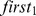 and 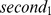, we have the function 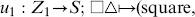 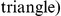, where 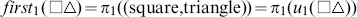 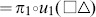. This function, 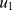, is the only function that satisfies the equality 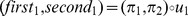, as required by the definition of product. Likewise, 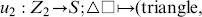 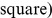 is the only function satisfying 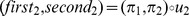.

In psychological/cognitive terms, the sets 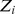, 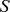 and 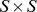 and the functions 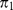, 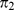 and 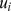 correspond to internal cognitive representational states and processes. The arrows 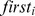 and 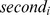 correspond to cognitive capacities derived from the composition of the other arrows. (The derived arrows representing computations are constructed from a *graph* representing sets of cognitive states—nodes—and processes—edges—by a functor sending each graph to the *free category* on that graph—add the identity arrow for each corresponding node and one arrow for each corresponding path consisting of more than one edge. A systematic relationship between cognitive and computational levels could be further developed in terms of *adjoint functors* [7], another kind of universal construction, denoted as the relation 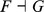, but that is beyond the scope of the current paper.) The collection of objects and arrows modeling the shape capacities is given in the following *commutative diagram* (i.e. paths from the same start object to the same end object are equal, where one path has at least two arrows)—sets and arrows associated with the 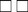 and 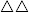 cases are not shown; a dashed arrow indicates uniqueness: 
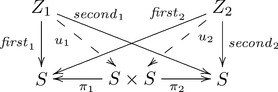
(1)


Systematicity is realized by common mediating functions 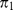 and 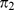, the presence or absence of each arrow implies the presence or absence of each collection of systematically related inferential capacities.

Our categorical explanation for F-systematicity appears to be analogous to the classical/connectionist explanation involving shared grammatical rules/weighted connections, which correspond to arrows in our terms. The critical difference, though, is the additional constraints imposed by the universal construction part of our explanation that is not present in a classical/connectionist explanation. A variation on the categorical architecture for the shapes example illustrates this difference.

Suppose we modify the categorical computational architecture that is shown in Diagram 1 by replacing the Cartesian product set 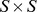 with the set 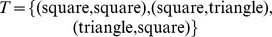. Accordingly, we also replace the projections 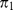 and 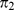 by modified projections 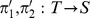 with element maps (respectively) that are the same as 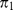 and 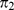, but without the corresponding element map for the pair 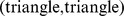 not in 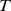. Similarly, the unique arrows 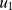 and 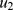 are replaced by arrows 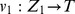 and 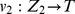 with element maps that are the same as 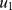 and 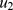 (respectively). The new architecture is shown in the following commutative diagram (where the arrows associated with 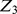 and the arrow 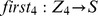 are not shown): 
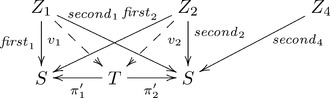
(2)


In this category, there does not exist an arrow 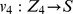 such that 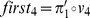 and 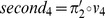. Note that the arrows 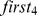 and 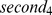 are given in this architecture, not derived from the composition of other arrows. Hence, the presence or absence of the arrows 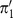 and 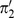 implies the presence or absence of all capacities 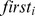 and 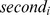 for 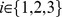 but not for 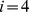. So, this architecture does not support systematicity even though it employs shared arrows 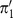 and 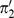. The critical difference here is that the construction 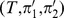 is not a universal construction, and so is excluded by our explanation of systematicity. As this example illustrates, our explanation for systematicity is neither a generalization nor a specialization of the classical one. Instead, our category theory explanation generalizes the classical one on the aspect of shared cognitive resources (generalizing from shared grammatical rules to shared categorical arrows), but specializes the classical explanation on the aspect of modes of compositionality (specializing arbitrarily available juxtapositioning of symbols to universally composable arrows). See [17] for a category theoretical approach to context-free languages.

#### F-systematicity, universal constructions and optimization.

The explanation for F-systematicity just provided emphasized universal constructions as they relate to equivalence classes. This perspective is natural given that F-systematicity pertains to equivalence classes of cognitive capacities. Another aspect of universal constructions is in relation to optimization. G-systematicity, that is the preference for connected over isolated relations in forming analogies, pertains to this aspect. Hence, we recast our explanation for F-systematicity in terms of the optimization aspect of universal constructions to prime a category-theoretic universal constructions approach to G-systematicity in analogy.

A universal construction is also a kind of optimization in the sense that it consists of an object (from a collection of objects) that is “closest” (relative to the collection) to an object of interest. If we consider each cognitive capacity as a path from one set of cognitive states (input) to another set of cognitive states (output), then closest is interpreted in terms of path length, i.e. the number of component arrows between two objects. The following diagram illustrates this conception by comparing the paths associated with the set 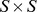 and the paths associated with the set 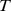, corresponding to architectures having universal and non-universal constructions (respectively): 
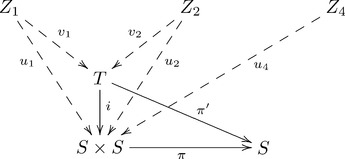
(3)


This diagram omits the functorial component of this universal construction and simplifies some objects and arrows to highlight the characterization of universal constructions as a kind of optimization. The omitted details are given in Text S1. In particular, the projection operators, 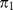 and 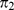, are simply denoted here as the arrow 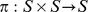, which stands in for the arrow pair 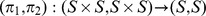. Likewise, 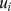 and 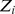 stand in for the arrow 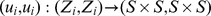, and similarly for 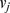 and 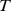. With the exception of 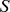, 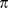 and 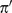, these objects and arrows belong to the *image* of the diagonal functor, sending objects and arrows to pairs of objects and arrows. The image of a functor 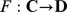 is the collection of objects 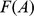 and arrows 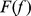.

Diagram 3 reveals the sense in which object 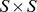 is closer to 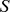 than 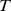. Arrow 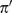 is composed of two arrows, the projection 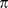 and the injection 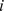. Hence, the “distance” (number of component arrows) from 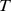 to 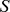 (two) is greater than the distance from 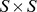 to 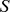 (one). All capacities 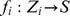 are composed of 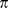, only capacities 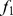, 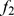 and 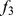 are composed of 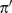. This conception of a universal construction as closeness motivates a reconceptualization of analogy as structure approximation to provide a categorical treatment of G-systematicity, next.

### G-systematicity (Gentner)

All categorical constructions, including universal constructions, reside in a category of some kind. So, the first step in providing a categorical account of G-systematicity is to recast source and target knowledge domains in terms of a suitable category. The second step is to show that G-systematicity derives from a universal construction in regard to that category. The explanation for G-systematicity also considers two cases of analogy: (1) a special case, where the source and target knowledge domains each consist of a single concept tree; and (2) the general case, where the source or target domains consist of multiple concept trees. Most (perhaps all) analogies concern the general case. However, the categorical explanation for the general case is a straightforward extension of the special case, hence this division of labor is also for didactic reasons. The formal details on which this account is based are provided in Text S2. The Water-Heat flow analogy (see Introduction) is used as an example application.

#### Special case: single pair of trees.

Recall that a category consists of objects, arrows and a composition operator for arrows satisfying certain axioms. Each of these components is defined in turn. In structure mapping theory, source/target knowledge is represented as tree-like concept structures. Here, each concept tree is considered as an object in some (to be specified) category. It is assumed that knowledge is represented in tree form, where every node has at most one parent. A concept that participates in two different relations will appear as two separate nodes, each representing the same concept but having a different parent, rather than a single node with two parents. In the water flow knowledge domain, for example, the binary relation *Contains*(*Vessel*, *Water*) is represented by the tree 
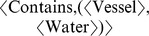
, where the first component is the relational concept (*Contains*) and the second component is the list of trees representing the related concepts (*Vessel* and *Water*). In general, an *n*-ary relation is represented by a tree consisting of an *n*-ary relational concept and a list of *n* concept trees. A limit of 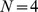 (i.e. quaternary trees) reflects a theoretical and empirical complexity limit of quaternary relations for adults [19]–[21].

The arrows in this category are specified next. Universal constructions can be conceptualized as a kind of optimization. Optimization suggests analogical mapping as involving a kind of structure approximation. Approximation ordering for inductively defined data structures, such as lists and trees, affords a categorical treatment of recursive computation [22]. The structures employed here are trees. Hence, approximation ordering over trees is considered as the arrows of this category of concept trees. In this context, approximation refers to partial knowledge about some concept tree. For example, suppose the contents of a vessel are unknown. A representation of this partial knowledge is the approximation tree 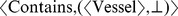, where the symbol 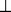 indicates the unknown concept tree. Conversely, for example, suppose one does not know the source of a water leak. This situation is represented by the tree 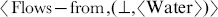.

Trees are (partially) ordered by an *approximation order relation*, denoted 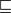. The expression 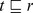 says that tree 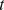 is no better an approximation (expresses no more knowledge) than a tree 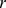; or, in passive form, tree 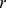 is at least as good an approximation (expresses at least as much knowledge) as tree 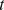. The definition of the specific approximation order relation for concept trees has two parts that formalize the following intuitions: (1) the concept tree 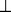 is no better an approximation (expresses no more knowledge) than any tree 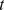, and (2) recursively, an *n*-ary tree 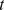 is no better an approximation (expresses no more knowledge) than an *n*-ary tree 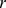 whenever the two trees express the same relational concept and each related tree 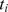 is no better an approximation than its corresponding related tree 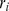. Formally, the approximation order relation for *n*-ary trees of arity 0 to 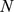 is defined by: 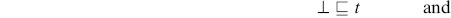


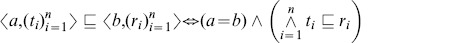



From the water flow knowledge domain, we have the following examples:

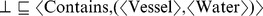
;

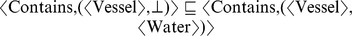
; and

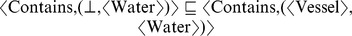
.

Some pairs of trees are not ordered, for example:

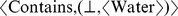
 and 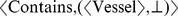; and

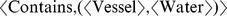
 and 
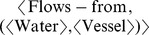
.
- The approximation order relation, 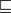, has three important properties (proved in Text S2).

1. *Reflexivity* (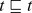): 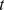 is no better an approximation than itself.
2. *Transitivity* (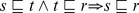): if 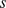 is no better an approximation than 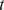, and 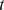 is no better an approximation than 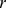 then 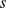 is no better an approximation than 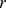.
3. *Antisymmetry* (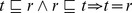): if 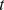 is no better an approximation than 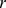, and 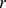 is no better an approximation than 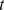 then 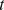 is the same as 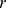.

Together, these three properties make the approximation relation, 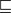, a *partial order*. The set of tree approximations, denoted 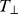, together with the partial order, 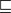, constitute a *partially ordered set*, or *poset*, denoted 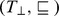.

Finally, the composition operator, 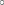, for partial orders is *conjunction* (of arrows). The transitivity property of partial orders means that if we have (the corresponding arrows) 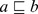 *and* 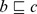 then we have (the arrow) 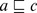, which satisfies the requirement for a category that there is an arrow for every pair of composable arrows. (Unlike *logical conjunction*, a conjunction of partial order arrows operator is not commutative, because 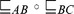 is undefined for 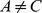.) The reflexivity property of partial orders means that for every element (object) 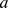 we have (the corresponding arrow) 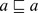, which satisfies the requirement for a category that every object has an identity arrow. The proof that this collection of objects, arrows and composition operator is a category follows immediately from the fact that 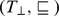 is a partially ordered set and every partially ordered set is a category.

We now want to consider a particular universal construction in regard to this category. Structure mapping theory says that analogical mapping involves identifying common relations between source and target domains, and G-systematicity is the principle that favours connected higher-order relations over isolated lower-order relations. Now that a category of concept trees and approximation orders is defined, we can consider an analogical mapping between two trees, 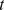 and 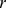, as involving their greatest common approximation tree, i.e. the tree 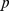 that shares the greatest number of (higher-order) relational concepts. Intuitively, the notion of a greatest common approximation tree is analogous to the concept of a greatest common divisor between numbers, or the greatest lower bound in a lattice. Indeed, category theory shows that these apparently similar concepts are formally instances of the same kind of construction, a product, which is a universal construction. Hence, our candidate construction is a product of concept trees in the category of trees and approximations.

The definition of the *greatest common approximation* (*gca*) of two trees is motivated by the following considerations:

- if either tree is the no approximation (no knowledge) tree, 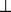, then their gca is also the no approximation tree;
- if either tree represents a different relational concept then their gca is also the no knowledge tree; and
- if both trees represent the same relational concept then their gca is that relational concept together with the gca of each pair of trees at the corresponding role of the relation.

Formally, the gca for trees 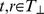 is defined by: 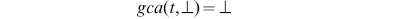


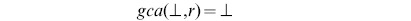


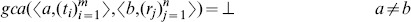


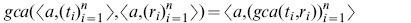



Some examples of the gca of two trees follow: 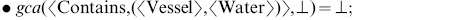


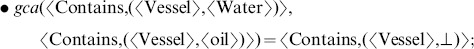


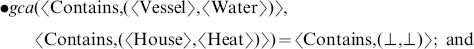


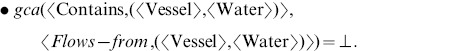



As the last example illustrates, gca is the greatest common approximation tree, not the correspondence between two trees: Contains and Flows-from are relational concepts, not relational concept trees, hence the greatest common approximation tree is not 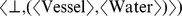. Having obtained the gca, a subsequent process can be employed to obtain correspondences between the other concepts (see also Discussion).

The gca of trees 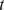 and 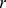 is their greatest lower bound 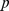. The poset 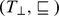 is a category where each tree 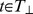 is an object in the (poset as a) category 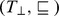. The product of trees 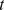 and 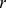 in this category is their gca together with two approximation arrows: i.e. 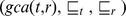. A proof is provided in Text S2. The proof follows from the proof that the gca of two trees is their greatest lower bound, and that the greatest lower bound is a product in a poset considered as a category.

The product of trees 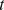 and 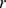 is the tree with the greatest number of connected higher-order relations in common to 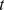 and 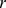 (together with their approximation arrows). A product is a universal construction. Hence, a universal construction provides an explanation for G-systematicity.

#### General case: multiple pairs of trees.

The explanation of G-systematicity in terms of universal constructions considered just a single pair of trees. In general, a source and target domain may consist of multiple trees, as illustrated in the Water-Heat flow example in the Introduction. The explanation generalizes to this situation. Here, we sketch the generalization.

Suppose there are multiple candidate pairs of source-target trees. The pairs of source-target trees considered during an analogy constitute a list of tree pairs. Computing the gca of each pair gives a list of product trees. Since product trees are also trees, we can also define an ordering on them. In this case, the ordering is over tree size, rather than tree approximation. Again, we have a partially ordered set and hence a product of product trees as the greatest lower bound. Suppose size is a natural number, for example, indicating tree height which corresponds to the order of the root relation, where size of the unknown tree is zero, i.e., 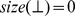. The set of natural numbers, 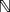, and the usual ordering on them, 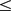, is the poset 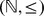. This set is also a *totally ordered set* (i.e. every pair of elements in the set is ordered) and the product of two natural number objects 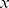 and 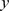 in the poset as a category 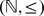 is the minimum of 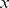 and 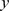: e.g., the *categorical* product 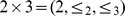. In the case that we require the maximum size of two trees, we can work in the dual (*opposite*) category 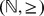, whose product is the maximum of two numbers: e.g., in 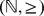, the *categorical* product 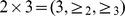. Equivalently, in 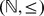, the maximum of two numbers is the dual, *coproduct* (universal) construction (denoted, 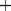): e.g., the *categorical* coproduct 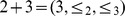.

Putting the two steps together (i.e. computing the gca for each pair of concept trees, and then computing the largest product tree) gives us the largest common approximation tree for the Water-Heat flow analogy, which corresponds to the G-systematicity principle. That is:


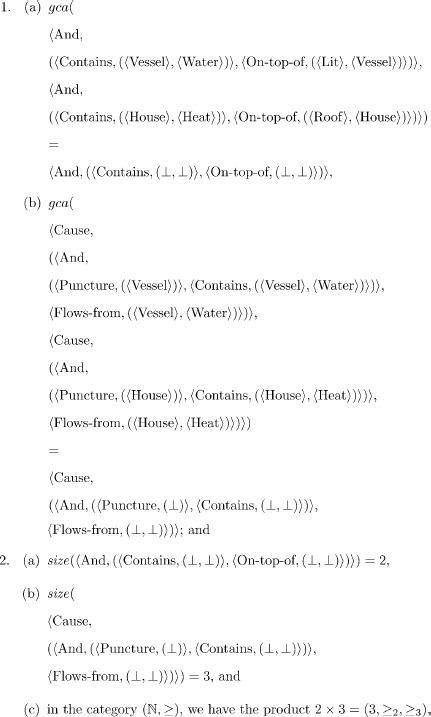


which corresponds to the largest approximation tree 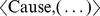.

Obtaining the largest tree in a list of 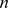 trees can be computed by an *n*-ary product, a product of 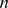 objects, which is a generalization of the binary products used to this point. However, an *n*-ary product may be psychologically implausible for large 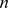 in the light of our account of cognitive complexity in terms of the arity of the underlying product [23]. Alternatively, we can provide a recursive definition of general products on an object 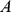, a set of concept trees, in terms of (at most) binary products. Informally, a general product is either an object 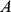 (unary product), or the binary product of 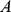 and a general product. In this way, we limit ourselves to binary products at every step, which is within the proposed capacity limits of older children and adults [23]. Pairs of trees are considered serially, in this case, instead of conjointly. The recursive approach also suggests another kind of universal construction. The details are beyond the scope of this paper, but algebras constructed on an *endofunctor* provide another kind of universal construction [14], [16] used in our explanation of systematically related recursively definable cognitive capacities [5]. (An endofunctor is a functor having the same domain and codomain category, hence its relevance to recursion.)

## Discussion

From a category theory perspective, F-systematicity and G-systematicity are two sides of the same coin; two aspects of a common principle, universal construction. Systematic cognitive capacity (F-systematicity) and analogical mapping of systems of higher-order relational concepts (G-systematicity) are hallmarks of human cognition. That they are two aspects of the same principle suggests that universal constructions (in the category-theoretic sense) are a crucial component of cognitive architecture.

Psychologically, we can consider universal constructions as a kind of optimization of cognitive resources. In the context of cognitive capacity, the F-systematicity property affords the ecological benefit of not having to expend further resources for an already present (component) cognitive capacity [3]. In the context of analogy, the G-systematicity property affords more correspondences between source and target knowledge domains [2], and therefore greater opportunities to exploit knowledge in one domain for inferences in another. Hence, systematic cognitive capacity and analogical mapping of systems of higher-order relations are two expressions of this one formal, optimization principle.

The explanation for G-systematicity involved two components: the gca trees computed from pairs of concept trees, and the largest gca from the list of computed gca trees. In terms of cognitive processes, one possible interpretation is that these two components reflect processes operating within and between a focus of attention. This interpretation is illustrated by the following sequence: two concept trees are in the current focus of attention; their gca is computed and stored in working memory; attention is shifted to a new pair of trees; the gca of the new pair is computed and compared to the gca already in working memory; if the gca tree of the new pair is larger than the one in working memory then the tree in working memory is replaced with the gca of the new pair; this process continues until some termination criterion at which point working memory holds the largest gca. How this category-theoretic level of analysis maps into a symbolic level, e.g., the *structure mapping engine* [24], or a connectionist level, e.g., DORA [25], of analysis is a further challenge. At the neural level, one approach that we have mentioned before [6] is to propose a suitable category of neural networks and arrows between networks. Networks are a kind of graph. The category of graphs and graph homomorphisms has products, so one possibility is a variation of this category that also has products.

A psychological interpretation of a categorical construction may also depend on the nature of the arrows and ambient category. The explanations for F-systematicity and G-systematicity involved one kind of universal construction (product), but two different types of arrows and hence categories: functions between sets for F-systematicity, and order relations between trees or numbers for G-systematicity. The interpretation of F-systematicity in terms of common processes mapping cognitive states to states is natural, but a similar interpretation for G-systematicity may appear less so, since the arrows appear to be comparisons (not transformations) between objects. Nonetheless, G-systematicity also has an interpretation in terms of common arrows: in a poset as a category, every comparison between objects 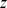 and 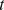 (and 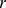), i.e. 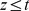, factors through the comparison of the least upper bound 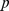 of 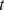 and 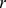, i.e. 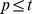, because the corresponding arrow 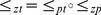 (i.e. 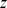 less-than-or-equal 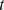 whenever 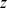 less-than-or-equal 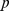 *and* 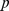 less-than-or-equal 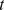). Hence, 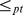 is the common arrow underlying all comparisons with 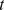, defining an equivalence class of capacities for comparisons with 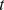 (and likewise 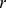). The general claim, then, is that these two forms of systematicity are expressions of a kind of optimization of cognitive resources.

### Summary and further directions

The category theory concept of a universal construction was introduced to address a limitation of the classical explanation for F-systematicity: i.e. the lack of an explanation as to why the grammars constituting cognitive architecture are just the systematic ones. The category theoretical explanation says that the (grammatical) structures supporting systematicity are the universal/optimal ones, in a precisely specified, formal sense. This category-theoretic principle of universal construction also accounts for G-systematicity as the same kind of optimal/universal structure, albeit in a different category.

Given the abstractness of category theory, one may wonder whether any collection of arrows (cognitive processes) can be characterized by a universal construction of some kind. In particular, any two cognitive processes as computable functions would seem to be characterizable by an architecture with the equivalent computational power of a universal Turing machine. In this case, every (computable) cognitive process is systematically related to every other (computable) cognitive process, which would seem to render the category theoretical explanation of systematicity as too powerful. However, the common property of computability is only one part of each process. (Recall that each arrow characterized by a universal construction is composed from a common mediating arrow and a unique arrow.) For unrelated cognitive processes, beyond the computability property in this example, there is no reason why having the unique component of one process implies having the unique component of the other process. This situation is the category theoretical analogue of knowing that *John* stands for the person John says nothing about knowing that *Mary* stands for the person Mary. An extreme example is where the mediating arrow is the identity arrow, which essentially means that the only thing two processes share is a common (co)domain. Hence, our universal constructions explanation does not imply some version of pan-systematicity. (Note also that not all categories have all kinds of universal constructions. For a simple example, a discrete category, having only identity arrows, does not have an *initial object* universal construction: i.e. for each object 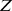 in the category there is a unique arrow from the initial object to 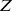.)

The category-theoretic explanation for G-systematicity is intended to complement models of analogy generally. Category theory was invented as a formal means of comparing mathematical structures so that the tools and techniques of one field may be carried over for the benefit of another [26]. Similarly, here, the additional value of revealing a connection between these two kinds of systematicity is the potential for exchange of methods and concepts for the mutual benefit of each discipline. For instance, F-systematicity is primarily a question about the cognitive structures that underlay collections of systematically related cognitive capacities, rather than the origins of those structures. Analogy research is also concerned with the induction of knowledge structures, such as addressed in the DORA model of analogy and schema induction [25]. Hence, methods and techniques used to address schema induction may also transfer to the F-systematicity domain for what is called *second-order systematicity* [10], the systematic capacity to *learn* certain cognitive capacities.

In the other direction, a categorical basis for F-systematicity of recursively definable cognitive capacities in terms of algebras constructed on an endofunctor [5], mentioned earlier, provides a unifying treatment of recursive computation generally [14]. Concept trees and products of them were defined recursively. Correspondences between the remaining concepts that are not common to both trees can also be computed recursively as the list consisting of a pair of corresponding relational concepts followed by the (possibly empty) list (in the case of nullary concept trees) of correspondences between their branch concept trees. Optimization has also been cast as a recursive computation (e.g., [27]). Hence, such algebras may also provide a unifying treatment for computational models of analogy.

There is a large literature on computational models of analogy for a broad range of phenomena (see [28] for a review), and the category theoretical approach presented here is a modest first step towards integrating properties of analogy with other components of cognition. One important aspect of analogy not addressed here is the role of the one-to-one correspondence principle that is a central feature of theories of analogy, such as structure mapping theory [2]. For example, the gca of *Causes*(*Loves*(*John*, *Mary*), *Kisses*(*John*, *Mary*)) and *Causes*(*Loves*(*Jane*, *Marcia*), *Kisses*(*Jane*, *Marcia*)) is the same as the gca of *Causes*(*Loves*(*John*, *Mary*), *Kisses*(*John*, *Mary*)) and *Causes*(*Loves*(*Jane*, *Marcia*), *Kisses*(*Susan*, *Tony*)), yet we may expect a preferential mapping to the first choice given that the repeating components (e.g., *John* as the lover and as the kisser) represent the same concept. One possibility is to include the dual notion of coproducts by considering each repetition as a single concept with more than one parent, i.e. by considering the structure as a lattice instead of a tree. In this case, matching is based on both top-down (product) and, dually, bottom-up (coproduct) universal constructions.

Another important aspect of analogy not addressed here is the semantic relatedness of concepts, which is addressed in models of analogy such as LISA [29] and DORA [25] using semantic feature units. For instance, these models prefer matching *Loves*(*John*, *Mary*) to *Likes*(*Bill*, *Susan*) than *Fears*(*Peter*, *Beth*), because *Loves* and *Likes* share more semantic features than *Loves* and *Fears* (see [29]). A categorical approach that combines syntactic (symbolic) and semantic (vectorial) aspects of language was proposed in [30], as a categorical product of corresponding components. A further challenge, then, is a category theoretical approach to integrating such syntactic and semantic aspects of analogy.

Category theory has further potential to reveal connections between cognitive components that may not be apparent from other theoretical approaches. To conclude with another example, it has been argued that the capacities for analogy and (relational) language are closely connected and unique to humans [31]. Elsewhere [5], we noted a distinction between kinds of recursive capacities based on the underlying endofunctor: e.g., the systematic capacities for recursion over numbers, lists and trees are based on universal constructions derived from endofunctors with different forms. In particular, tree-related algebras involve a more “complex” endofunctor than list-related algebras, analogous to the difference between quadratic and linear functions. The connection between analogy and language may depend on systematic capacities for recursion that are tied to tree-related algebras and the common “quadratic” form of their underlying endofunctor. Having such an endofunctor and associated universal construction affords the capacity for analogy if and only if the capacity for language as another kind of systematicity. Such formal, category-theoretic connections hint at a further deepening of our understanding of the structure of human cognition.

## Supporting Information

Text S1 **Basic category theory definitions and examples used in support of a category theoretical treatment of the two kinds of systematicity.**
(PDF)

Text S2 **Category theory definitions, examples, propositions and theorems pertaining to concept trees and G-systematicity.**
(PDF)
