# Supplementary material for: Analogy, Cognitive Architecture and Universal Construction: A Tale of Two Systematicities
Source: PLoS One. 2014 Feb 25;9(2):e89152. doi: 10.1371/journal.pone.0089152 (PMC3934878; doi:10.1371/journal.pone.0089152)
Supplement: Text S1 — Basic category theory definitions and examples used in support of a category theoretical treatment of the two kinds of systematicity. (PDF) [file pone.0089152.s001.pdf]

Text S1

Basic category theory definitions, examples, propositions and theorems are provided in this appendix. The intention here is to provide sufficient formal support for the arguments in the main text, rather than a complete introduction to category theory. Deeper and broader introductions to category theory can be found in many books on the subject (see, e.g., [1, 2]).

Definition (Category). A *category* $\mathbf{C}$ consists of:

- a class of *objects* $|\mathbf{C}| = (A, B, \ldots)$;
- for each pair of objects A and B in $\mathbf{C}$, a set $\mathbf{C}(A, B)$ of *arrows*, also called *morphisms*, or *maps*, from A to B, called a *hom-set*, where each arrow $f : A \to B$ has A as its *domain* and B as its *codomain*, including the *identity arrow* $1_A : A \to A$ for each object A; and
- a *composition operation*, denoted $\circ$, of arrows $f : A \to B$ and $g : B \to C$, written $g \circ f : A \to C$, that satisfies the axioms of:
  - *associativity*: $(h \circ g) \circ f = h \circ (g \circ f)$, for all $f : A \to B,\ g : B \to C,\ h : C \to D$; and
  - *identity*: $1_B \circ f = f = f \circ 1_A$, for all $f : A \to B$. □

Remark (Composable). An arrow f is said to be *composable* with an arrow g whenever the codomain of f equals the domain of g. The composition of f *with* g is the arrow $g \circ f$. □

Remark (Arrow, morphism, map). Arrow, morphism and map are synonymous, though usage varies and is not strict. Here we use: arrow, generally; morphism to emphasize additional structure, e.g., *universal morphism* includes object and arrow components; and map to emphasize the action on elements for arrows between sets, e.g., map $f : A \to B; a \mapsto b$ sends element $a \in A$ to element $b \in B$. □

Example (Set). The category **Set** has sets for objects and total functions for arrows, where the identity arrows are the identity functions and composition is function composition. □

Example (Poset). A *partially ordered set* (*poset*) is a set P together with a binary relation $\leq$ on P, denoted as the pair $(P, \leq)$, that is *reflexive* ($p \leq p$), *transitive* ($p \leq q \wedge q \leq r \Rightarrow p \leq r$) and *antisymmetric* ($p \leq q \wedge q \leq p \Rightarrow p = q$). A *monotonic function* is a function $f : P \to Q$ such that $a \leq b \Rightarrow f(a) \leq f(b)$ for all $a, b \in P$. The category **Poset** has posets for objects and monotonic functions for arrows, with identity arrows and composition as for **Set**. □

Example (Discrete category). A *discrete category* is a category that has no non-identity arrows. A set A is construed as a discrete category by regarding each element $a \in A$ as an object and associating with each a an identity arrow, $1_a : a \to a$. □

Example (Poset as category). A poset $(P, \leq)$ is a category whose objects are the elements $p, q \in P$ with one arrow $p \to q$ just in case $p \leq q$. □

Example (Pointed set, $\mathbf{Set}_\perp$). A *pointed set*, denoted $P_\perp$, is a set P with a designated element $\perp$, called the *point* of the set. A point-preserving function is a total function $f : P_\perp \to Q_\perp$ that sends the point $\perp_P \in P_\perp$ to the point $\perp_Q \in Q_\perp$. The category of pointed sets, $\mathbf{Set}_\perp$, has pointed sets for objects and point-preserving functions for arrows, with identity arrows and composition as for **Set**. □

Remark (Partial function). The category **Pfn** of sets and partial functions is equivalent to the category $\mathbf{Set}_\perp$ of pointed sets and total (pointed) functions, where the point, $\perp$, assumes the role of undefined element. □

Definition (Product). In a category $\mathbf{C}$, a *product* of objects A and B is an object P, also denoted $A \times B$, together with two arrows $p_1 : P \to A$ and $p_2 : P \to B$, conjointly denoted (P, p_1, p_2), such that for every object $Z \in |\mathbf{C}|$ and every pair of arrows $f : Z \to A$ and $g : Z \to B$ there is a unique arrow $u : Z \to P$, also denoted as $\langle f, g \rangle$, such that $f = p_1 \circ u$ and $g = p_2 \circ u$, as indicated in commutative diagram

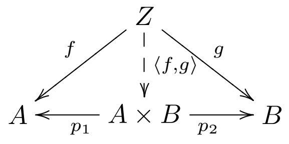

(1)

□

Example (Cartesian product). A *Cartesian product* of sets A and B, denoted $A \times B$ is the set of all pairs of elements with the first (second) element taken from A (B), i.e. $A \times B = \{(a, b)|a \in A, b \in B\}$, together with two functions (projections) for retrieving the first and second elements of each pair, i.e., $\pi_1 : A \times B \to A; (a, b) \mapsto a$, $\pi_2 : A \times B \to B; (a, b) \mapsto b$. A Cartesian product is a product in **Set**. □

Example (Greatest lower bound). In a poset $(P, \leq)$, a *lower bound* of an element $p \in P$ is an element $w \in P$ such that $w \leq p$. A *greatest lower bound* (*glb*) of elements $p, q \in P$ (if it exists) is a lower bound r of p and q such that for every lower bound z of p and q, we have z is a lower bound of r (i.e. $r \leq p \wedge r \leq q$ and for all $z \in P$ we have $z \leq p \wedge z \leq q \Rightarrow z \leq r$). A greatest lower bound is a product in a poset as a category. □

Definition (Functor). A *functor* from a category $\mathbf{C}$ to a category $\mathbf{D}$ is a map $F : \mathbf{C} \to \mathbf{D}$ that sends each object A in $\mathbf{C}$ to an object $F(A)$ in $\mathbf{D}$, and each arrow $f : A \to B$ in $\mathbf{C}$ to an arrow $F(f) : F(A) \to F(B)$ in $\mathbf{D}$, satisfying the following axioms:

- *compositionality*: $F(g \circ_{\mathbf{C}} f) = F(g) \circ_{\mathbf{D}} F(f)$ for all arrows $f : A \to B$ and $g : B \to C$ in $\mathbf{C}$; and
- *identity*: $F(1_A) = 1_{F(A)}$ for each object A in $\mathbf{C}$. □

Example (Diagonal functor). A *diagonal functor*, denoted Δ, sends each object A to its pair (A, A) and each arrow $f : A \to B$ to its pair (f, f). That is, $\Delta : \mathbf{C} \to \mathbf{C} \times \mathbf{C}; A \mapsto (A, A), f \mapsto (f, f)$. □

Example (Product functor). A *product functor*, denoted Π, sends each pair of objects (A, B) to their product object $A \times B$ and each pair of arrows (f, g) to their product arrow $f \times g$. That is, $\Pi : \mathbf{C} \times \mathbf{C} \to \mathbf{C}; (A, B) \mapsto A \times B, (f, g) \mapsto f \times g$. □

With the definitions of category and functor, we are ready to define universal construction. A universal construction comes in two varieties: *universal morphism* and *couniversal morphism*, which is the *dual* construction and obtained by reversing the directions of the arrows in the definition of universal morphism. We just provide the definition of universal morphism.

Definition (Universal morphism). Given a functor $F : \mathbf{A} \to \mathbf{C}$ and an object $Y \in |\mathbf{C}|$, a *universal morphism* from F to Y is a pair consisting of an object A in $\mathbf{A}$, and a arrow ϕ in $\mathbf{C}$, denoted (A, ϕ), such that for every object $Z \in |\mathbf{A}|$ and every arrow $f : F(Z) \to Y$, there exists a unique arrow $u : Z \to A$, such that $\phi \circ F(u) = f$, as indicated in commutative diagram

$$\begin{array}{ccccc} Z & & F(Z) & & \\ {\scriptstyle u}\downarrow & & {\scriptstyle F(u)}\downarrow & \searrow{\scriptstyle f} & \\ A & & F(A) & \xrightarrow[\phi]{} & Y \end{array} \tag{2}$$

□

Example (Product as universal morphism). A product $(A \times B, (p_1, p_2))$ is a universal morphism, and indicated in commutative diagram

$$\begin{array}{ccccc} Z & & (Z, Z) & & \\ {\scriptstyle \langle f,g\rangle}\downarrow & & {\scriptstyle (\langle f,g\rangle,\langle f,g\rangle)}\downarrow & \searrow{\scriptstyle (f,g)} & \\ A \times B & & (A \times B, A \times B) & \xrightarrow[(p_1,p_2)]{} & (A, B) \end{array} \tag{3}$$

where functor $F : \mathbf{A} \to \mathbf{C}$ in the definition of universal morphism is the diagonal functor, and product object $A \times B$ is obtained by application of the product functor to object (A, B). Compare Diagram 3 to Diagram 1. □

Definition (Universal construction). A *universal construction* is either a universal morphism, or a couniversal morphism. □

Example (Product as universal construction). A product is a universal construction. □

References

1. Mac Lane S (2000) Categories for the working mathematician. Graduate Texts in Mathematics. New York, NY: Springer, 2nd edition.

2. Simmons H (2011) An introduction to category theory. New York, NY: Cambridge University Press.
