# Supplementary material for: Analogy, Cognitive Architecture and Universal Construction: A Tale of Two Systematicities
Source: PLoS One. 2014 Feb 25;9(2):e89152. doi: 10.1371/journal.pone.0089152 (PMC3934878; doi:10.1371/journal.pone.0089152)
Supplement: Text S2 — Category theory definitions, examples, propositions and theorems pertaining to concept trees and G-systematicity. (PDF) [file pone.0089152.s002.pdf]

Text S2

In this section, we define a (pointed) set of concept trees and a partial (approximation) ordering over them, and together they constitute a poset and hence a category. Moreover, a product in this category is a greatest lower bound on the approximation of two concept trees. This categorical approach provides the formal basis for deriving Gentner's systematicity from universal constructions.

Definition (n-ary relational concept). An *n-ary relational concept* is a concept that takes n concepts as related arguments. Constants or features are regarded as 0-ary (nullary) relational concepts. Predicates are regarded as 1-ary (unary) relational concepts. Binary relations are regarded as 2-ary, ternary relations as 3-ary, quaternary as 4-ary relational concepts, etc. □

Example (n-ary relational concepts). Nouns and values, e.g., *ball*, *red*, are typically 0-ary relational concepts, as they do not take any concepts as relational arguments. Categories of features, e.g., *colour*, are usually 1-ary relational concepts, as they take a single argument as the related concept, e.g., *colour red*. *Loves* is a binary relational concept, e.g., *John loves Mary*. *Gives* is a ternary relational concept, e.g., *John gives Mary a book*. □

Definition (N-ary relational concept trees). Let A_n be a set of n-ary relational concepts, for $0 \leq n \leq N$. A *relational concept tree* is a pair consisting of a single n-ary relational concept ($a \in A_n$) *leaf* and a sequence of n relational concept *branch* trees, denoted $(t_1, \ldots, t_n)$, or in shorter index form, $(t_i)_{i=1}^n$. A relational concept tree is denoted $\langle a, (t_1, \ldots, t_n)\rangle$, or $\langle a, (t_i)_{i=1}^n\rangle$. When $n = 0$ (a constant concept tree), the sequence of branch trees is empty, denoted $()$, and in a slight abuse of notation, a constant concept tree $\langle a, ()\rangle$ is simply denoted $\langle a\rangle$. An *N-ary relational concept tree* is a relational concept tree with maximum relational concept arity N over all subtrees. □

Example (N-ary relational concept trees). An example of a 0-ary (constant) relational concept tree is $\langle \text{John}\rangle$. An example of a 1-ary relational concept tree is $\langle \text{colour}, (\langle \text{red}\rangle)\rangle$. An example of a 2-ary relational concept tree is $\langle \text{loves}, (\langle \text{John}\rangle, \langle \text{Mary}\rangle)\rangle$.

Definition (Relational concept order). The *relational concept order* of a concept in a concept tree is either: zero for 0-ary relational concepts; or one plus the maximum order of the concepts in its branch trees for i-ary concepts, $1 \leq i$. Concepts of order one are called *first-order*, and concepts of order greater than one are called *higher-order* relational concepts. □

Example (Relational concept order). *Loves* is a first-order concept in $\langle \text{loves}, (\langle \text{John}\rangle, \langle \text{Mary}\rangle)\rangle$. *Knows* is a higher(second)-order concept in the tree $\langle \text{knows}, (\langle \text{Sue}\rangle, \langle \text{loves}, (\langle \text{John}\rangle, \langle \text{Mary}\rangle)\rangle)\rangle$. □

Definition (Pointed tree). A *pointed tree* is an N-ary relational concept tree constructed from a set of relational concept trees that includes a designated point element $\perp$. The point is the "unknown" concept tree. □

Example (Pointed tree). An example is $\langle \text{loves}, (\langle \text{John} \rangle, \perp) \rangle$, which indicates that what is loved by John is not known. □

Definition (Approximation ordering). The *approximation ordering* $\sqsubseteq$ is a binary relation on a set of N-ary relational (approximation) concept trees, $T_\perp$, that is defined for $0 \leq n \leq N$ by:

$$\perp \sqsubseteq t \qquad \text{and}$$

$$\langle a, (t_i)_{i=1}^n \rangle \sqsubseteq \langle b, (r_i)_{i=1}^n \rangle \Leftrightarrow (a = b) \wedge \left(\bigwedge_{i=1}^n t_i \sqsubseteq r_i \right)$$

□

Example (Tree orderings). The following are examples of approximation orderings on pointed trees: $\perp \sqsubseteq \perp$; $\perp \sqsubseteq \langle \text{John} \rangle$; and $\langle \text{loves}, (\langle \text{John} \rangle, \perp) \rangle \sqsubseteq \langle \text{loves}, (\langle \text{John} \rangle, \langle \text{Mary} \rangle) \rangle$. Examples of pairs of trees that are not ordered are: $\langle \text{John} \rangle$ and $\langle \text{Mary} \rangle$; and $\langle \text{loves}, (\langle \text{John} \rangle, \perp) \rangle$ and $\langle \text{loves}, (\perp, \langle \text{Mary} \rangle) \rangle$. □

Remark (Least). $\perp$ is the *least* element of $T_\perp$, i.e. $\perp \sqsubseteq t$ for all $t \in T_\perp$. □

Proposition (Approximation partial order). The approximation ordering $\sqsubseteq$ is a partial order.

Proof. We are required to prove that $\sqsubseteq$ is: *reflexive*, $t \sqsubseteq t$; *transitive*, $s \sqsubseteq t \wedge t \sqsubseteq r \Rightarrow s \sqsubseteq r$; and *antisymmetric*, $t \sqsubseteq r \wedge r \sqsubseteq t \Rightarrow t = r$ for all $s, t, r \in T_\perp$. The proof is by *structural induction.* Structural induction proceeds analogously to mathematical induction. First, we prove that the target proposition holds for the base case. Next, we prove that if the target proposition holds for trees $(t_i)_{i=1}^n$, then it holds for $\langle a, (t_i)_{i=1}^n \rangle$.

Reflexive:

Base ($t = \perp$): Immediate, since $\perp \sqsubseteq \perp$ (from the definition).

Hypothesis ($(t_i)_{i=1}^n$): For each i, we assume $t_i \sqsubseteq t_i$.

Induction ($\langle a, (t_i)_{i=1}^n \rangle$): Immediate (from the definition), since $a = a$ and $t_i \sqsubseteq t_i$ (by hypothesis) implies $\langle a, (t_i)_{i=1}^n \rangle \sqsubseteq \langle a, (t_i)_{i=1}^n \rangle$.

Transitive:

Base $(s, t, r = \bot)$:

$$\begin{aligned} s \sqsubseteq t \wedge\ t \sqsubseteq r \quad & \text{(definition)} \\ \Rightarrow \bot \sqsubseteq \bot \quad & \text{(substitution)} \\ \Rightarrow s \sqsubseteq r \quad & \end{aligned}$$

Hypothesis $((s_i)_{i=1}^n, (t_i)_{i=1}^n, (r_i)_{i=1}^n)$: For each i, we assume the following holds: $s_i \sqsubseteq t_i \wedge t_i \sqsubseteq r_i \Rightarrow s_i \sqsubseteq r_i$.

Induction $(\langle a, (s_i)_{i=1}^n\rangle, \langle b, (t_i)_{i=1}^n\rangle, \langle c, (r_i)_{i=1}^n\rangle)$:

$$\begin{aligned} & \langle a, (s_i)_{i=1}^n\rangle \sqsubseteq \langle b, (t_i)_{i=1}^n\rangle \wedge\ \langle a, (s_i)_{i=1}^n\rangle \sqsubseteq \langle c, (r_i)_{i=1}^n\rangle && \text{(definition)} \\ & \Rightarrow (a = b) \wedge (s_i \sqsubseteq t_i)_{i=1}^n \wedge (b = c) \wedge (t_i \sqsubseteq r_i)_{i=1}^n && \text{(=, hypothesis)} \\ & \Rightarrow (a = c) \wedge (s_i \sqsubseteq r_i)_{i=1}^n && \text{(definition)} \\ & \Rightarrow \langle a, (s_i)_{i=1}^n\rangle \sqsubseteq \langle c, (r_i)_{i=1}^n\rangle && \end{aligned}$$

Antisymmetric:

Base $(t, r = \bot)$: Immediate.

Hypothesis $((t_i)_{i=1}^n, (r_i)_{i=1}^n)$: For each i, we assume the following proposition holds: $t_i \sqsubseteq r_i \wedge r_i \sqsubseteq t_i \Rightarrow t_i = r_i$.

Induction $(\langle a, (t_i)_{i=1}^n\rangle, \langle b, (r_i)_{i=1}^n\rangle)$:

$$\begin{aligned} & \langle a, (t_i)_{i=1}^n\rangle \sqsubseteq \langle b, (r_i)\rangle \wedge\ \langle b, (r_i)\rangle \sqsubseteq \langle a, (t_i)_{i=1}^n\rangle && \text{(definition)} \\ & \Rightarrow (a = b) \wedge (t_i \sqsubseteq r_i)_{i=1}^n \wedge (r_i \sqsubseteq t_i)_{i=1}^n && \text{(hypothesis)} \\ & \Rightarrow (a = b) \wedge (t_i = r_i)_{i=1}^n && \text{(equality)} \\ & \Rightarrow \langle a, (t_i)_{i=1}^n\rangle = \langle b, (r_i)_{i=1}^n\rangle && \end{aligned}$$

□

Proposition (Tree category). A pointed set of N-ary relational concept trees $(T_\bot)$ together with an approximation ordering $(\sqsubseteq)$ is a category.

Proof. Immediate from the fact that $(T_\bot, \sqsubseteq)$ is a poset. □

Definition (gca). The function (binary operator) $gca : T_\bot \times T_\bot \to T_\bot$, which produces the *greatest*

common approximation of N-ary relational concept trees, is defined for all $t, r \in T_\perp$ and $0 \leq m, n \leq N$ by:

$$\begin{aligned} gca(t, \perp) &= \perp \\ gca(\perp, r) &= \perp \\ gca(\langle a, (t_i)_{i=1}^m \rangle, \langle b, (r_j)_{j=1}^n \rangle) &= \perp && a \neq b \\ gca(\langle a, (t_i)_{i=1}^n \rangle, \langle a, (r_i)_{i=1}^n \rangle) &= \langle a, (gca(t_i, r_i))_{i=1}^n \rangle \end{aligned}$$

□

Example (gca). The gca of $\langle \text{loves}, (\langle \text{John} \rangle, \perp) \rangle$ and $\langle \text{loves}, (\perp, \langle \text{Mary} \rangle) \rangle$ is $\langle \text{loves}, (\perp, \perp) \rangle$. □

Theorem (gca). The greatest common approximation of trees $t, r \in T_\perp$ is the greatest lower bound of t and r.

Proof. By structural induction: we need to show that for all $z, t, r \in T_\perp$ when $z \sqsubseteq t \wedge z \sqsubseteq r$ we have $z \sqsubseteq gca(t, r)$. In short, we need to prove the following target proposition: $z \sqsubseteq t \wedge z \sqsubseteq r \Rightarrow z \sqsubseteq gca(t, r)$.

Base ($t = \perp$): The only case of z for which $z \sqsubseteq \perp \wedge z \sqsubseteq r$ is $z = \perp$, since $\perp$ is the least element of $T_\perp$. Hence, we have:

$$\begin{aligned} z \sqsubseteq \perp \wedge z \sqsubseteq r \Rightarrow z \sqsubseteq gca(t, r) && \text{(substitution)} \\ \Rightarrow \perp \sqsubseteq gca(t, r) && \text{(least element)} \\ \Rightarrow \text{True} && \text{(reduction)} \\ \text{True} && \end{aligned}$$

Hypothesis ($(t_i)_{i=1}^n$): For each i, we assume the following proposition holds: $z_i \sqsubseteq t_i \wedge z_i \sqsubseteq r_i \Rightarrow z_i \sqsubseteq gca(t_i, r_i)$.

Induction ($\langle a, (t_i)_{i=1}^n \rangle$): By definition (approximation), there are two cases of z for which $z \sqsubseteq \langle a, (t_i)_{i=1}^n \rangle$: (1) $z = \perp$ and (2) $z = \langle a, (z_i)_{i=1}^n \rangle$, where $z_i \sqsubseteq t_i$.

Case 1: This case has already been proven in the base case, $t = \perp$.

Case 2: Given that $z = \langle a, (z_i)_{i=1}^n \rangle$, the target proposition only requires us to consider the trees r where

$\langle a, (z_i)_{i=1}^n \rangle \sqsubseteq r$. By definition (approximation), we only need to consider the trees $r = \langle a, (r_i)_{i=1}^n \rangle$, where $z_i \sqsubseteq r_i$. Hence, given $z = \langle a, (z_i)_{i=1}^n \rangle$, $r = \langle a, (r_i)_{i=1}^n \rangle$ and $z_i \sqsubseteq r_i$, we are required to prove:

$$z \sqsubseteq \langle a, (t_i)_{i=1}^n \rangle \wedge z \sqsubseteq r \rangle \Rightarrow z \sqsubseteq gca(\langle a, (t_i)_{i=1}^n \rangle, r \rangle)$$

(substitution)

$$\Rightarrow \langle a, (z_i)_{i=1}^n \rangle \sqsubseteq gca(\langle a, (t_i)_{i=1}^n \rangle, \langle a, (r_i)_{i=1}^n \rangle)$$

(definition of *gca*)

$$\Rightarrow \langle a, (z_i)_{i=1}^n \rangle \sqsubseteq \langle a, (gca(t_i, r_i))_{i=1}^n \rangle$$

(definition of *approximation*)

$$\Rightarrow \bigwedge_{i=1}^{n} z_i \sqsubseteq gca(t_i, r_i)$$

(hypothesis)

$$\Rightarrow \text{True}$$

(reduction)

True

□

Theorem (Tree product, gca). In the (pointed poset as a) category $(T_\perp, \sqsubseteq)$, a product of trees $t, r \in T_\perp$ is the greatest common approximation tree of t and r together with the two approximation arrows, $\sqsubseteq_t$: $t \times r \to t$ and $\sqsubseteq_r$: $t \times r \to r$, together denoted $(t \times r, \sqsubseteq_t, \sqsubseteq_r)$.

Proof. Immediate from the fact that the greatest common approximation tree is the greatest lower bound, a greatest lower bound is a product in a pointed poset as a category, and $(T_\perp, \sqsubseteq)$, as a pointed poset, is a category. □

Remark (All products). The category $(T_\perp, \sqsubseteq)$ is said to have *all* binary products, since gca (hence, product) is defined for all pairs of trees in $T_\perp$. □
